# Heparin Induces Harmless Fibril Formation in Amyloidogenic W7FW14F Apomyoglobin and Amyloid Aggregation in Wild-Type Protein *In Vitro*

**DOI:** 10.1371/journal.pone.0022076

**Published:** 2011-07-13

**Authors:** Silvia Vilasi, Rosalba Sarcina, Rosa Maritato, Antonella De Simone, Gaetano Irace, Ivana Sirangelo

**Affiliations:** 1 Dipartimento di Biochimica e Biofisica, Seconda Università ` degli Studi di Napoli, Naples, Italy; 2 Istituto Nazionale Biostrutture e Biosistemi, Rome, Italy; 3 Dipartimento di Medicina Pubblica Clinica e Preventiva, Seconda Università di Napoli, Naples, Italy; Illinois Institute of Technology, United States of America

## Abstract

Glycosaminoglycans (GAGs) are frequently associated with amyloid deposits in most amyloid diseases, and there is evidence to support their active role in amyloid fibril formation. The purpose of this study was to obtain structural insight into GAG-protein interactions and to better elucidate the molecular mechanism underlying the effect of GAGs on the amyloid aggregation process and on the related cytotoxicity. To this aim, using Fourier transform infrared and circular diochroism spectroscopy, electron microscopy and thioflavin fluorescence dye we examined the effect of heparin and other GAGs on the fibrillogenesis and cytotoxicity of aggregates formed by the amyloidogenic W7FW14 apomyoglobin mutant. Although this protein is unrelated to human disease, it is a suitable model for *in vitro* studies because it forms amyloid-like fibrils under physiological conditions of pH and temperature. Heparin strongly stimulated aggregation into amyloid fibrils, thereby abolishing the lag-phase normally detected following the kinetics of the process, and increasing the yield of fibrils. Moreover, the protein aggregates were harmless when assayed for cytotoxicity *in vitro*. Neutral or positive compounds did not affect the aggregation rate, and the early aggregates were highly cytotoxic. The surprising result that heparin induced amyloid fibril formation in wild-type apomyoglobin and in the partially folded intermediate state of the mutant, i.e., proteins that normally do not show any tendency to aggregate, suggested that the interaction of heparin with apomyoglobin is highly specific because of the presence, in protein turn regions, of consensus sequences consisting of alternating basic and non-basic residues that are capable of binding heparin molecules. Our data suggest that GAGs play a dual role in amyloidosis, namely, they promote beneficial fibril formation, but they also function as pathological chaperones by inducing amyloid aggregation.

## Introduction

Amyloid diseases are related to anomalies in the folding process of certain proteins that may form insoluble fibril deposits. They include over 20 clinically relevant disorders, among which neurodegenerative disorders such as Alzheimer's disease, and non neuropathic conditions such as type-II diabetes [1]. Amyloid fibrils share common structural features despite the considerable diversity in the primary sequence of the constituent proteins. They are rich in β-sheet structures and the ordered regions adopt the classic cross-β structure in which individual strands in the β-sheets run perpendicular to the long axis of the fibril with the inter β-sheet hydrogen bonds oriented parallel to the fibril axis [2]–[4]. A wide range of proteins and peptides that do not form amyloid *in vivo* can be induced to do so *in vitro* and this has led to the hypothesis that the ability to form amyloid is a general property of polypeptide chains [3]. Amyloid fibril formation in bulk solution occurs through a nucleation-dependent polymerization process consisting of two phases, i.e., nucleation and extension. The initial step of nucleus formation consists in the association of monomers. This process is thermodynamically unfavorable and is the rate-limiting step of the fibrillation process. Once a nucleus has formed, the further addition of monomers to the nucleus becomes thermodynamically favorable and results in rapid extension of amyloid fibrils *in vitro* [5]. The exact nature of the pathogenic amyloid species is matter of intense debate, but there is increasing evidence that oligomers or intermediates rather than fibrils are responsible for cytotoxicity and the associated cell death in amyloid diseases [6]–[8]. One therapeutic option is to design small molecules to block aggregation or to stabilize benign oligomers formed on or off the amyloid formation pathway [9], [10]. However, there is evidence that promoting the formation of insoluble aggregates could lower the concentration of the toxic oligomers or intermediates associated with disease and thus protect against damage [11]–[13].

Recently, attention has focused on the effect of the biological environment in which aggregation occurs naturally. In fact, the biological milieu can profoundly influence the mechanism and rate of process, as well as the structure and stability of the resulting fibrils [14]. In particular, considerable effort has been devoted to clarifying the role of glycosaminoglycans (GAGs) in protein aggregation. Structurally, GAGs are a group of negatively charged heterogeneous polysaccharides resulting from the assembly of repeating disaccharide units and are one of the main components of the extracellular matrix [15], [16]. In most amyloid diseases, GAGs are often associated with amyloid deposits, and there is evidence that they play an active role in favoring amyloid fibril formation and stabilization [17]–[20]. Snow and Kisilevsky [21] reported an increase in GAG levels at the time of serum amyloid A deposition. More recently, it was demonstrated that inhibition of heparan sulfate biosynthesis is directly correlated with loss of amyloid deposition in amyloid A animal models [22]–[24]. Evidence for the relation between GAGs and amyloid comes also from *in vitro* studies. GAGs stimulate, *in vitro*, the formation of amyloid fibrils from the Alzheimer Aβ protein [25]–[27]; heparin and, to a lesser extent, heparan sulfate have been reported to increase significantly the rate of fibrillation of tau protein, α-synuclein, gelsolin, AcP, β2-microglobulin and the aortic amyloid polypeptide medin [28]–[34]. Heparan sulfate has also been found to convert the prion protein from the PrP^C^ to the PrP^SC^ form [35]. Generally, among GAGs, heparin is particularly effective in accelerating fibril formation probably because of its high content of sulfate groups [25]. Several studies have demonstrated that electrostatic interactions are important in the binding of heparin to amyloid fibrils. In particular, removal of all sulfate groups from heparin or the addition of magnesium or calcium ions suppresses these interactions, thereby indicating their electrostatic nature [25], [32]. Moreover, it has been postulated that the amyloid-promoting activity of heparin is facilitated through specific amyloid polypeptide-heparin interactions via binding sites [36]–[43].

Notwithstanding the large body of data associating heparin and other GAGs with amyloidogenesis, little is known about the mechanism by which heparin promotes amyloid formation or about its effect on the overall aggregation pathway. It has been supposed that, similarly to catalyzed reactions, GAGs favour aggregation, nucleation and amyloid fibril formation by a mechanism substantially different from that occurring in bulk solution [44]. The data available suggest that heparin can influence and promote the misfolding of polypeptides into proamyloidogenic intermediates rich in β-sheet and may also function as a structural template for self-assembly. Recent studies on acetyl phosphatase have shown that heparin sulfate induces changes in the aggregation process by splitting it in a parallel manifold faster pathway [45]. Moreover, heparin accelerates transthyretin (TTR) aggregation by a scaffold-based mechanism in which the sulfate groups interact primarily with TTR oligomers through electrostatic interactions, by concentrating and orienting the oligomers and, subsequently, facilitating the formation of higher molecular weight aggregates [46].

We previously showed that a mutated form of apomyoglobin, i.e., W7FW14F, undergoes a nucleation-dependent polymerization reaction that results in the formation of amyloid fibrils identical to those formed by proteins involved in amyloid diseases [47], [48]. Although the W7FW14F apomyoglobin mutant is unrelated to any human disease, it is a suitable model for amyloid aggregation studies because it forms amyloid-like fibrils under physiological conditions of pH and temperature. Under these experimental conditions, wild-type apomyoglobin is in the globular, α-helical native state. In the present study, we used W7FW14F apomyoglobin to study the effect of GAGs, mainly heparin and related compounds on the various kinetic phases of W7FW14F apomyoglobin amyloid aggregation. The results show that both the extent and rate of formation of amyloid fibrils are greatly enhanced by heparin and certain other GAGs, but not by the neutral and positively charged polymers dextran and polylysine. The amyloid aggregates formed in the presence of heparin appear to be harmless. Thus, heparin eliminates cytotoxic oligomeric species by promoting the formation of benign fibrils. Interestingly, we found that heparin induces early toxic aggregates also in wild-type apomyoglobin (a protein that does not show any tendency to aggregate). This could open a debate regarding the therapeutic use of heparin.

## Materials and Methods

### Materials and Protein Purification

All chemicals were purchased from Sigma Chemical Co (St. Louise, MO). The GAGs and polymers used were: porcine intestinal heparin (grade I-A, molecular weight 18000 and 5000 Da), heparan sulfate (HS), chondroitin sulfate A, B and C (ChonA, DS and ChonC), dextran and dextran sulfate, polylysine (Mw 70000-15000) and polyarginine (Mw 15000-70000). Wild-type and W7FW14F apomyoglobin were expressed and purified as described previously [46]. Protein concentration was measured under denaturing conditions by UV absorption using an ε_280_ value of 13,500 M^−1^ cm^−1^ for wild-type and an ε_275_ value of 3750 M^−1^ cm^−1^ for W7FW14F apomyoglobin.

### Fibril Formation

Aggregation of W7FW14F apomyoglobin was initiated by raising the pH of a 40 µM protein solution from 4.0 to 7.0. This results in the formation of protein aggregates in a prefibrillar form that turn into mature fibrils in 7–15 days [48]. GAGs were added to the protein solution both before the pH was raised to neutrality, and immediately after. The effect of GAGs was identical in both conditions.

### Spectroscopic measurements

Absorption measurements were recorded at 25°C on a Jasco V-550 double-beam spectrophotometer. The protein samples, at a concentration of 40×10^−6^ M, were centrifuged at 20000 *g* for 30 min and the absorbance at 280 nm of supernatant solution was measured. A single-exponential function was fitted to the kinetic plots of the measured absorbance versus time to determine the apparent aggregation rate constants. The following equation was used:
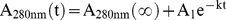
(1)where A_280 nm_(∞) is the limiting absorbance, A_1_ and K are the amplitude and rate constant of the observed change, respectively.

Far UV circular dichroism (CD) spectra were recorded at 25°C on a Jasco J-810 spectropolarimeter using thermostated quartz cells of 0.1 cm. Spectra were acquired at 0.2-nm intervals with a 4 s integration time and a bandwidth of 1.0 nm. An average of three scans was obtained for all spectra. Photomultiplier absorbance did not exceed 600 V in the spectral region analyzed. Data were corrected for buffer contributions and smoothed using the software provided by the manufacturer (System Software version 1.00). All measurements were performed under nitrogen flow. The protein samples (40×10^−6^ M) were diluted 1∶2 before spectra acquisition. The results were expressed as mean residue ellipticity [Θ]_MRW_ in units of degree cm^2^ dmol^−1^.

### Thioflavin T fluorescence measurements

The aggregation kinetics was monitored using the dye Thioflavin T (ThT) that exhibits enhanced fluorescence upon binding to amyloid fibrils. Fluorescence measurements were carried out with a Perkin Elmer Life Sciences LS 55 spectrofluorimeter. Excitation and emission wavelengths were set at 450 and 482 nm, respectively. The excitation and emission slit widths were set at 5 nm each. ThT stock solution was prepared in Tris buffer (pH 8.0, 20 mM) at a concentration of 500 µM and stored at 4°C. At different time intervals, aliquots of samples (40 µM), incubated in the presence or in the absence of GAGs, were mixed (1∶1 v/v) with buffer containing ThT. The final ThT concentration was 50 µM. The fluorescence spectra were recorded and the fluorescence intensity at 482 nm was corrected by subtracting the emission intensity of ThT/GAGs solutions.

### Fourier transform infrared measurements

Fourier Transform Infrared (FTIR) spectra were recorded on a Multiscope FT-IR microscope coupled with a Spectrum One spectrometer (PerkinElmer, Wellesley, MA, USA). The FTIR spectra in transmission mode were collected (4000 cm^−1^-600 cm^−1^ range) at a resolution of 4 cm^−1^ with 16 accumulations per run. For each spectrum, signals corresponding to the water and CO_2_ vapors were automatically subtracted and the baseline corrected. Spectra were recorded with dry samples of protein obtained from repeated cycles of lyophilization and dissolution in D_2_O at a concentration of 40 µM.

### Transmission electron microscopy

Fibril formation in the presence of heparin was monitored by transmission electron microscopy (EFTEM Lybra 120, Zeiss, Germany). Protein aliquots of 10 µL were sampled from a protein solution of 40 µM, diluted 1∶10 and deposited on 400-mesh formvar-coated grid (Electron Microscopy Sciences, Hatfield, UK) and allowed to absorb for about 1 min. The excess liquid was removed with filter paper. A drop of negative stain (1% aqueous uranyl acetate made up fresh (Laurylab, Saint-Fons Cedex, France) was placed on the grid for 1 min and allowed to dry.

### Cell culture and incubation with protein aggregates

NIH-3T3 cells (mouse fibroblasts, American Type Culture Collection) were cultured in Dulbecco's modified eagle's medium (DMEM)-high glucose supplemented with 10% bovine calf serum and 3.0 mM glutamine in a 5.0% CO_2_ humidified environment at 37°C. 50 units/mL penicillin and 50 µg/mL streptomycin were added to the medium. The cells were plated at a density of 100,000 cells/well on 12-well plates in 1 mL of medium. After 24 h, protein samples (40 µM) were mixed 1∶1 v/v with cell media and incubated. Cells in culture medium without protein served as control.

### MTT assay

Cell viability was assessed as the inhibition of the ability of cells to reduce the metabolic dye 3-[4,5-dimethylthiazol-2-yl]-2,5-diphenyltetrazolium bromide (MTT) to a blue formazan product [49]. After 24 h of incubation with protein samples, cells were rinsed with phosphate buffer solution (PBS). 100 µL of a stock MTT solution (5 mg/mL in PBS) were then added to 900 µL of DMEM without phenol red containing 10% bovine calf serum/well, and incubation was continued at 37°C for an additional 3 h. The medium was aspirated, and cells were treated with isopropyl alcohol-0.1 M HCl for 20 min. Levels of reduced MTT were determined by measuring the difference in absorbance at 570 and 690 nm. Data are expressed as average percent reduction of MTT with respect to the control ± S.D. from five independent experiments carried out in triplicate. For statistical analysis, we used a two-tailed Student's *t* test with unequal variance at a significance level of 5% unless otherwise indicated.

## Results

### The effect of heparin on aggregation process of W7FW14F apomyoglobin at pH 7.0

The effect of heparin as modulator of the amyloid aggregation of the W7FW14F apomyoglobin mutant was explored using absorbance, CD, FTIR, ThT fluorescence and EM measurements. We previously showed that, similar to the wild-type protein, the W7FW14F mutant is fully unfolded at pH 2.0 and partially folded at a pH near 4.0. When pH is increased from 4.0 to 7.0, the mutant protein aggregates and forms amyloid fibrils by a characteristic nucleation-dependent polymerization mechanism, whereas the wild-type protein folds correctly [47], [48]. At the beginning of the aggregation process, the aggregating protein molecules have a native-like conformation with an abundant alpha-helical content. These species assemble to form oligomeric species that, between 12 and 24 h, give rise to amyloid-like protofilaments. The latter develop slowly to form, after 5–6 days, protofibrils that then associate further to form the higher order amyloid fibrils. The early aggregates have the structural characteristics of amyloid precursors, namely, the ability to bind ThT and Congo red, and an extensive β-sheet structure [48], [50].

**Figure 1:**
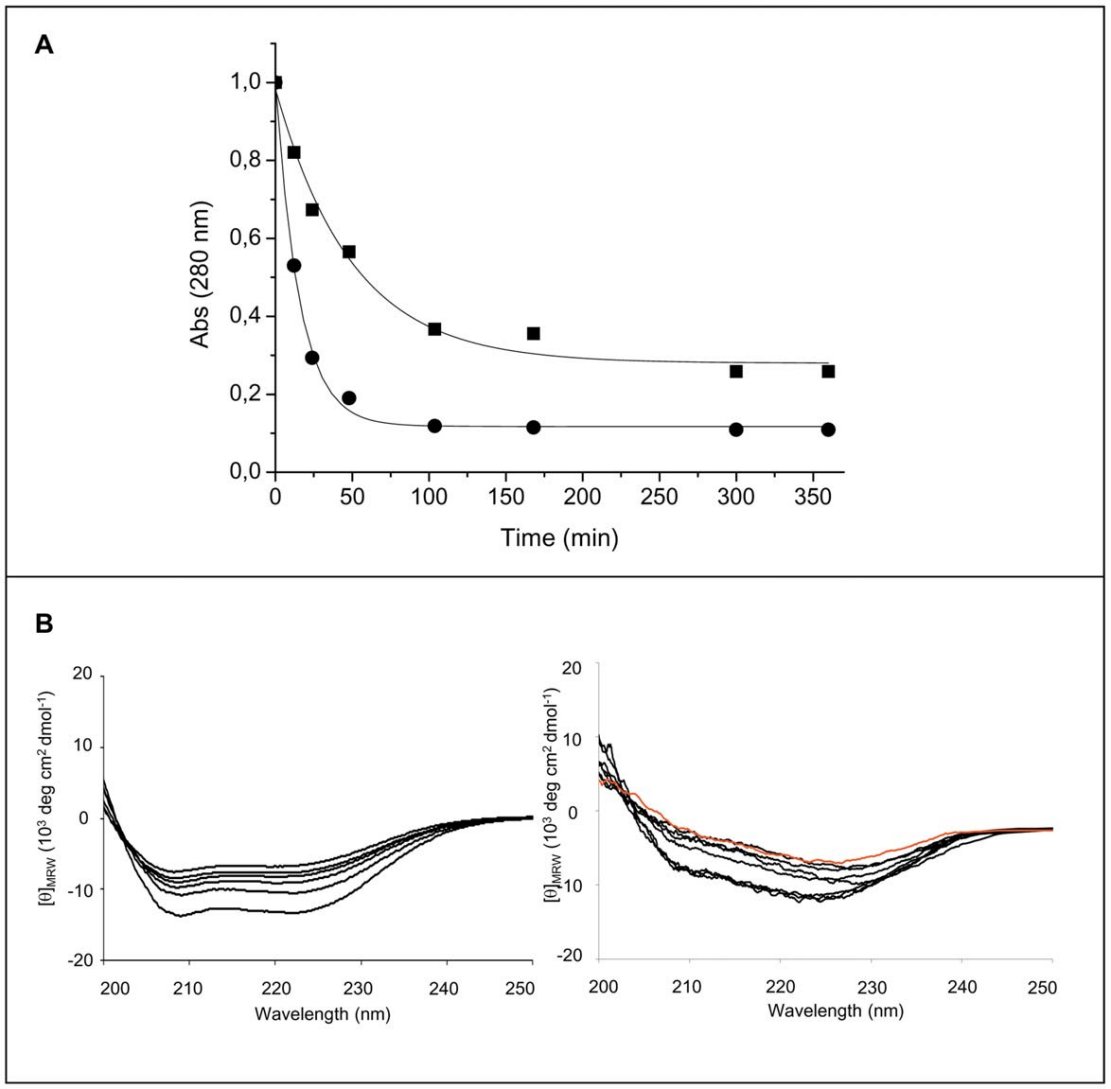
Effect of heparin on the early stage of the aggregation process of W7FW14F apomyoglobin at pH 7.0. **Panel A**: Aggregation was monitored by measuring the absorbance at 280 nm of supernatant solution after centrifugation in the absence (▪) and in the presence of 0.1 mg/mL heparin (•). Protein concentration was 40 µM. Points are experimental values, continuous lines were obtained from an exponential fit as A_280 nm_(t) = A_280 nm_(∞)+A_1_ e^−kt^. **Panel B**: Time-dependence of the far-UV CD spectra in the absence (left) and in the presence (right) of 0.1 mg/mL heparin. From the lower to the upper spectrum, times are: 1, 5, 20, 120, 240, and 360 min. The protein samples (40 µM) were diluted 1∶2 before spectra acquisition. In red is displayed the spectrum recorded at the end of fibrillation process in the absence of heparin.

We first studied the effect of heparin on the early stage of the aggregation process by measuring the protein concentration of the soluble fraction upon increasing the pH from 4.0 to 7.0. Heparin was added to protein solution both immediately before and after the pH was increased to neutrality. Protein samples were then centrifuged at 20,000 *g* for 30 min to obtain a supernatant prevalently containing soluble protein, and absorbance was measured at 280 nm. Figure 1A shows the time-dependence of W7FW14F apomyoglobin aggregation at pH 7.0 in the presence and absence of heparin. The more rapid decrease of absorbance at 280 nm observed in the presence of heparin indicates that the aggregation rate increased upon GAG addition. The aggregation rates obtained by fitting the experimental data to a single exponential function A_280 nm_(t) = A_280 nm_(∞)+A_1_ e^−kt^ (equation 1) were: 0.014±0.0035 min^−1^ and 0.056±0.006 min^−1^ in the absence and in the presence of heparin, respectively. Moreover, the fraction of soluble protein was greatly reduced in the presence of heparin, which suggests that heparin promoted the formation of a higher amount of aggregates.

To gain further insight into the mechanism by which heparin increased the aggregation rate of the amyloid-forming apomyoglobin mutant, we analyzed the far-UV CD spectra as a function of time. Circular dichroism spectra were recorded immediately after pH neutralization of the protein samples (Figure 1B). During the first 6 h of observation, in the absence of heparin (left), the shape of the CD spectra did not change, all spectra showing the two minima at 208 and 222 nm, which is characteristic of an α-helical conformation, whereas the intensity of the CD signal decreased gradually due to the increase of light scattering caused by protein aggregation [50]. The CD spectrum was very different in the presence of heparin (right). In fact, soon after the pH was increased to neutrality, the minimum centered at 222 nm was still evident whereas that at 208 nm was very faint and disappeared as aggregation proceeded. The spectrum recorded after completion of the fibril formation process of the W7FW14F apomyoglobin in the absence of heparin is shown for comparison purposes.

**Figure 2:**
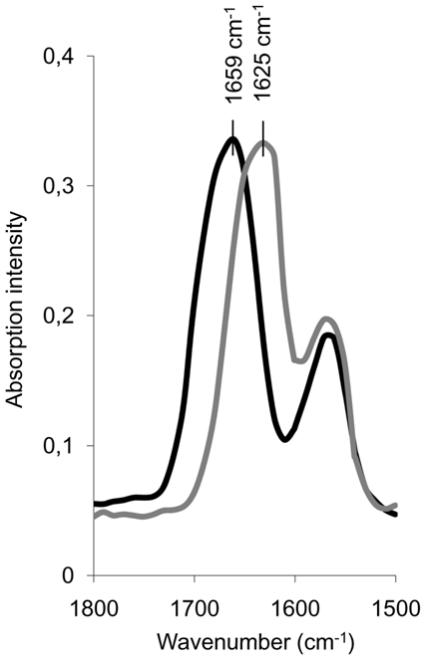
FTIR spectra in the amide I' region of amyloid-forming W7FW14F apomyoglobin at pH 7.0. The spectra have been recorded at the beginning of the aggregation process in the absence (black line) and in the presence (gray line) of 0.1 mg/mL heparin.

We used FTIR spectroscopy, which is a very sensitive technique widely used to monitor the α-to *β*-transition underlying amyloid formation [51], [52], to obtain direct information about the structure of W7FW14F apomyoglobin aggregates formed in the presence of heparin. Figure 2 shows the spectra recorded soon after the onset of the aggregation reaction performed with and without heparin. The spectrum recorded in the absence of heparin shows an amide I' at 1659 cm^−1^, which indicates that the first aggregates formed contain a considerable amount of α-helical conformation, and that the beta transition has not yet occurred [50]. Conversely, the spectrum recorded in the presence of heparin has an amide I' maximum close to 1625 cm^−1^. This value is typical of an amyloid conformation [53] and is similar to that found for the amide I' of amyloid fibrils formed by apomyoglobin [50], [52].

**Figure 3:**
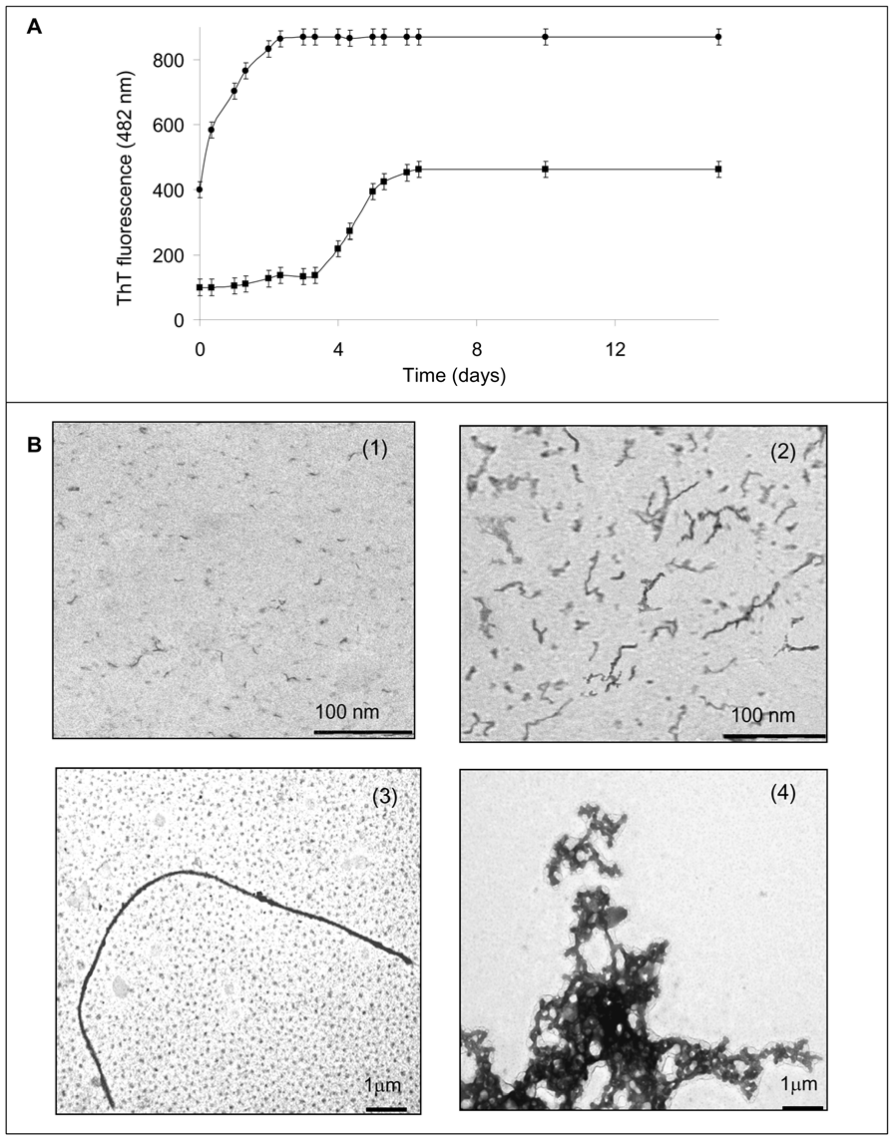
Effect of heparin on the fibrillogenesis process of W7FW14F apomyoglobin at pH 7.0. **Panel A**: ThT fluorescence in the absence (▪) and in the presence (•) of 0.1 mg/mL heparin. Protein concentration was 40 µM. Other experimental details are described in Materials and Methods. **Panel B**: Electron microscope images of W7FW14F apomyoglobin in the absence of heparin at the beginning (1) and at the end of the aggregation process (3), and in the presence of heparin at the beginning of the aggregation process (2) and 2–4 days thereafter (4).

We used ThT fluorescence to follow the time course of fibril formation. This dye forms a complex with the cross-β structure and its fluorescence intensity increases proportional to the amount of fibrils present when the ThT concentration is held constant [54], [55]. As shown in Figure 3A, in the absence of heparin, ThT fluorescence exhibited a sigmoidal time course, with a lag phase of approximately 4 days, as reported previously [48]. Heparin dramatically accelerated the amyloid aggregation process. In fact, it caused an instantaneous increase in ThT fluorescence intensity thereby determining loss of the lag-phase in the amyloid fibril formation kinetics. Moreover, the plateau fluorescence value was much higher with than without heparin. The addition of salts (200 mM NaCl) to protein samples incubated with heparin resulted in an aggregation kinetics superimposable to that observed in the absence of heparin (data not shown), thus suggesting that the heparin-induced acceleration of W7FW14F apomyoglobin aggregation is due largely to electrostatic interactions between the two oppositely charged molecules.

Using electron microscopy, we next examined the morphology of the W7FW14F apomyoglobin aggregates obtained in the absence and presence of heparin at the onset of aggregation (Figure 3B). As expected, only granular species were observed in the absence of heparin, whereas protofibrils were found in the heparin-treated sample. Consistent with FTIR spectra and ThT staining, the electron microscope images show that the formation of fibrillar species is accelerated in the presence of heparin. However, the EM images recorded 2–4 days after the onset of aggregation revealed that the fibrillar species obtained in the presence of heparin are branched and thicker than mature fibrils of control protein at the end of aggregation process (Figure 3C). The same picture was obtained at longer times.

**Figure 4:**
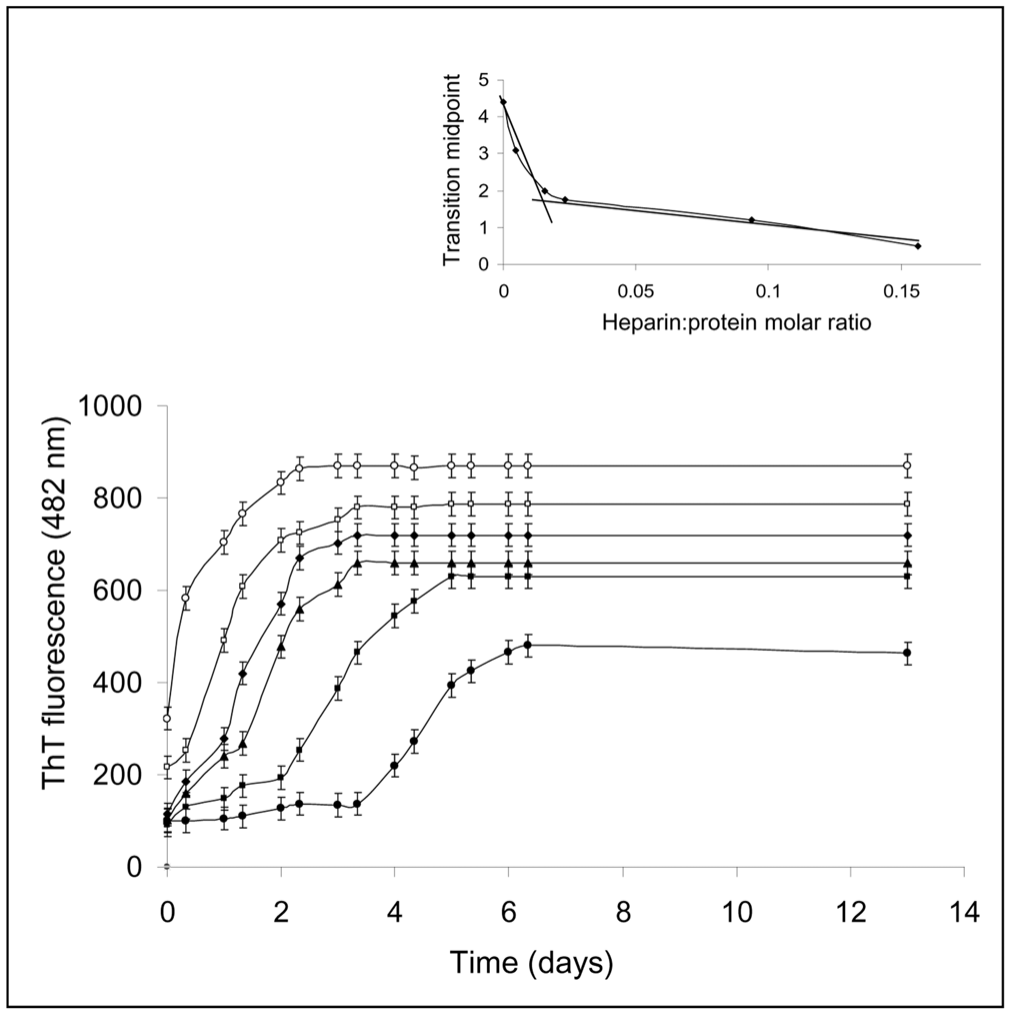
Effect of heparin concentration on W7FW14F apomyoglobin fibrillation kinetics. Fibrillization was monitored by the increase in fluorescence of ThT, as described under Materials and Methods. Protein concentration was 40 µM. Heparin concentrations were 0.1 (○), 0.06 (□), 0.015 (♦), 0.010 (▴), 0.003 (▪), and 0 mg/mL (•).The inset shows the dependence of the transition midpoint on heparin/apomyoglobin molar ratio.

We also evaluated whether the fibrillation kinetics was related to heparin concentration to understand better the effect of heparin on the lag phase. The addition of increasing concentrations of heparin led to a reduction of the lag and a progressive increase in the magnitude of the fluorescence plateau value (Figure 4). The dependence of the transition midpoint on the heparin/apomyoglobin molar ratio (inset of figure) indicates that the number of W7FW14F apomyoglobin molecules per heparin molecule varies from 40 to 5 on increasing polyanion concentration. In summary, incubation of W7FW14F apomyoglobin with heparin resulted in a substantial acceleration of the aggregation and fibrillation processes, and the magnitude of the acceleration effect was related to GAG concentration.

### The effect of heparin on the aggregation process of W7FW14F apomyoglobin at pH 4.0

**Figure 5:**
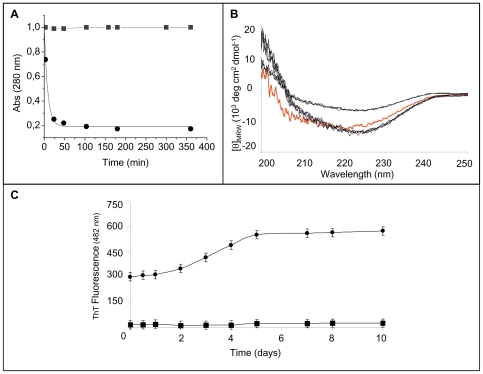
Effect of heparin on W7FW14F apomyoglobin at pH 4.0. **Panel A**: Absorbance at 280 nm of supernatant solution in the absence (▪) and in the presence of 0.1 mg/mL heparin (•). Protein concentration was 40 µM. Points are experimental values, continues line was obtained from an exponential fit as A_280 nm_(t) = A_280 nm_(∞)+A_1_ e^−kt^. **Panel B**: Time-dependence of the far-UV CD spectra in the presence of 0.1 mg/mL heparin. From the lower to the upper spectrum, times are: 20, 40, 60, 120, 180, 300 and 360 min. The protein samples (40 µM) were diluted 1∶2 before spectra acquisition. In red is displayed the spectrum recorded in the absence of heparin. **Panel C**: ThT fluorescence in the absence (▪) and in the presence of 0.1 mg/mL heparin (•). Protein concentration was 40 µM. Other experimental details are reported under “Materials and Methods”.

To probe further the mechanism by which heparin induced amyloid formation, we investigated its effect on the partially folded soluble conformation that W7FW14F apomyoglobin adopts near pH 4.0 [47]. Under this condition, the addition of heparin caused aggregation (Figure 5A) with a kinetics rate faster than that observed at pH 7.0, i.e., 0.096±0.015 min^−1^ vs. 0.056±0.006 min^−1^. The CD spectra recorded during the first 6 h of the process (Figure 5B) revealed the presence of two distinct successive conformational populations. The first appeared soon after the addition of heparin (20, 40, 60, and 120 min), whereas the second appeared much later (3, 5, and 6 h). It is interesting to note that the CD spectra obtained soon after the addition of heparin at pH 4.0 are similar, if not identical, to those recorded at neutral pH in the presence of heparin, whereas the spectra obtained at longer times are practically identical with that of mature fibrils (Figure 1B). FTIR analysis revealed that the amide I' maximum of both forms is close to 1625 cm^−1^ (data not shown), which supports the concept that the aggregated protein is essentially β-structured. Moreover, the heparin-induced aggregates formed at pH 4.0 were also able to bind ThT (Figure 5C). The intensity of ThT fluorescence measured soon after heparin addition was three-fold higher than that detected at pH 7.0 in the absence of heparin, which indicates that the aggregates have a prevalently cross-β structure, consistent with FTIR analysis. The initial instantaneous appearance of ThT fluorescence was followed by further increases and reached maximum value 5 days after the addition of heparin.

To examine the effect of heparin on fully unfolded apoprotein, we carried out the same experiments at pH 2.0 in the presence of heparin. The results were superimposable to those obtained at pH 4.0 thereby showing that heparin is also able to induce aggregation of unfolded apomyoglobin (data not shown).

### The cytotoxicity of heparin-induced W7FW14F apomyoglobin aggregates

**Figure 6:**
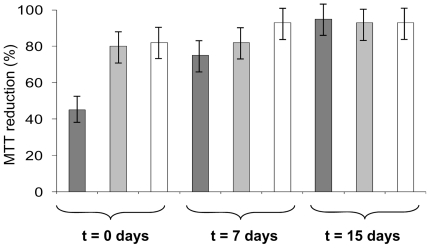
Effect of W7FW14F apomyoglobin aggregates on cell viability detected by MTT assay. NIH-3T3 cells were exposed to aggregates formed in the absence (dark grey) and in the presence of 0.1 mg/mL heparin at pH 7.0 (light grey) and at pH 4.0 (white). Aliquots of protein were taken at 0, 7, and 15 days from the onset of fibrillogenesis and incubated for 24 h with cells. Data are expressed as average percentage of MTT reduction ± SD relative to cells treated with medium alone or medium plus heparin, from triplicate wells from 5 separate experiments. Other experimental details are reported under “Materials and Methods”. Only early aggregates formed in the absence of heparin caused a significant decreases in reduced MTT levels (*P*<0.01).

We assessed the cytotoxicity of W7FW14F apomyoglobin aggregates obtained in the presence of heparin using the MTT assay, which is a rapid and sensitive indicator of amyloid-mediated toxicity. The toxicity of amyloid aggregates is closely linked to the presence of oligomeric species; in fact, decreased levels of MTT reduction are usually detected in the presence of soluble prefibrillar oligomers but not of fibrils [48], [56]–[60]. We compared the toxicity of aggregates formed in the absence and presence of heparin at aggregation onset and 7 and 15 days thereafter, at both pH 4.0 and pH 7.0. As expected, in the absence of heparin, early prefibrillar aggregates were toxic (about 45% MTT reduction), whereas fibrillar aggregates (at 7 and 15 days) were not (Figure 6) in accordance with a previous study [48]. The aggregates formed by the protein in the presence of heparin, at both pH 7.0 and 4.0, did not affect cell viability even at the beginning of the aggregation process. The lack of cytotoxicity well correlates with our finding that heparin induces rapid formation of harmless fibrillar aggregates.

### The effect of heparin on wild-type apomyoglobin

**Figure 7:**
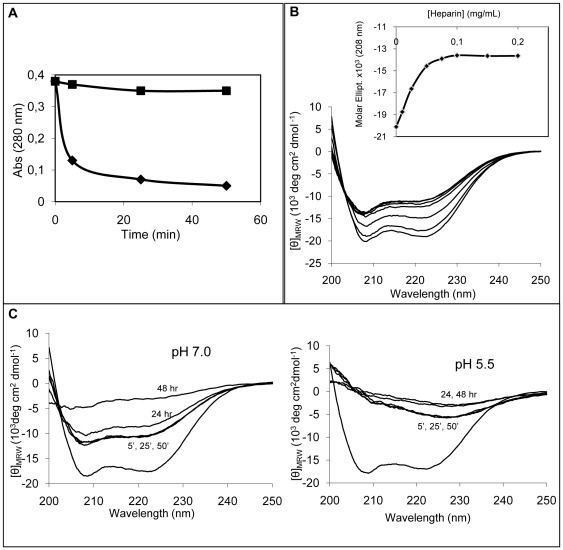
Effect of heparin on wild-type apomyoglobin at pH 7.0 and 5.5. **Panel A**: Absorbance at 280 nm of supernatant solution in the presence of 0.1 mg/mL heparin at pH 7.0 (▪) and at pH 5.5 (♦). Protein concentration was 40 µM. Points are experimental values, continues lines were obtained from an exponential fit as A_280 nm_(t) = A_280 nm_(∞)+A_1_ e^−kt^. **Panel B**: Effect of increasing concentration of heparin on the far-UV CD spectra of protein at pH 7.0. From the lower to the upper spectrum, heparin concentrations were: 0, 0.01, 0.025, 0.05, 0.075, 0.1 and 0.2 mg/mL. The protein samples (40 µM) were diluted 1∶2 before spectra acquisition. The inset shows the dependence of the molar ellepticity at 208 nm on heparin concentration. **Panel C**: Time-dependence of CD spectra in the presence of 0.1 mg/mL of heparin at pH 7.0 (left) and pH 5.5 (right). The lower spectra are those of protein in the absence of heparin.

The addition of heparin to native wild-type apomyoglobin caused protein aggregation (Figure 7). The rate of aggregation depended on pH in the range 5.5–7.0, at which the protein retains its native fold. At neutral pH, heparin induced only a slight turbidity, whereas massive precipitation was observed at lower pH values. Figure 7A shows the time course of protein aggregation measured at pH 7.0 and 5.5 in the first hour after the addition of heparin. At pH 5.5, the fraction of precipitated apomyoglobin was much higher than that measured at pH 7.0 (88% versus<10%). The time course of protein aggregation at pH 5.5 was not affected by 200 mM NaCl.

Figure 7B shows the effect of increasing heparin concentration on the CD spectrum of wild-type apomyoglobin at neutral pH. Heparin progressively reduced the intensity of the signal, which reached a plateau at a concentration as high as 0.1 mg/mL (inset of Figure 7B). Next, we examined the time-dependence of the CD spectrum of wild-type apomyoglobin in the presence of 0.1 mg/mL heparin at pH 7.0 and pH 5.5 (Figure 7C). The CD spectra drastically changed upon the addition of heparin, and remained stable for 1 h thereafter. Major changes were observed after 24 and 48 h. In particular, the CD spectrum recorded at pH 5.5 48 h after heparin addition was very similar, if not identical, to that recorded upon completion of the fibrillation process of the amyloid-forming mutant in the absence of heparin. The secondary structure content of the species appearing at different times and pH values was estimated and the results are reported in Table 1.

**Table 1:** Secondary structure percent content of wild-type apomyoglobin at various pHs and heparin incubation times.

pH	Time	Hep*	α	β	Turn	Random
7.0	0	−	0.58	0.06	0.13	0.23
7.0	25 min	+	0.34	0.16	0.20	0.30
7.0	24 h	+	0.27	0.22	0.21	0.30
7.0	48 h	+	0.08	0.36	0.22	0.34
5.5	25 min	+	0.20	0.37	0.20	0.23
5.5	24 h	+	0.05	0.45	0.22	0.28
5.5	48 h	+	0.05	0.45	0.21	0.29
6.0	25 min	+	0.35	0.17	0.20	0.28

*Heparin: 0.1 mg/mL.

It is feasible that the above results are related to the extent of histidine protonation that occurs in the pH range examined. As pH decreases, the net positive charge of the protein molecule increases, thereby favoring interactions with the negatively charged groups of heparin. The interaction between heparin and apomyoglobin is also responsible for the occurrence of conformational changes revealed by the extensive variation of the shape of CD spectra and by the related CD analysis of secondary structure shown in Table 1.

**Figure 8:**
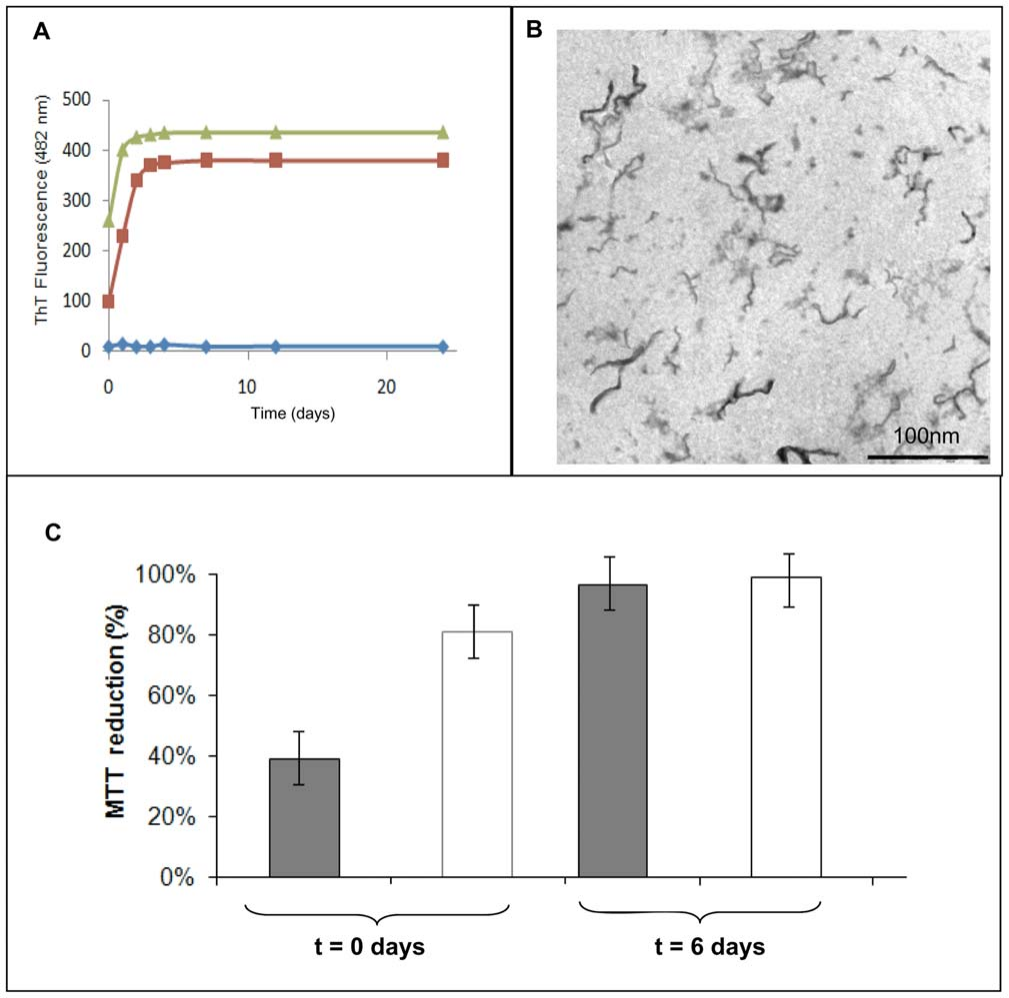
Effect of heparin on wild-type apomyoglobin amyloid aggregation and cytotoxic activity. **Panel A**: ThT fluorescence in the absence (♦) and in the presence of 0.1 mg/mL heparin at pH 5.5 (▴) and at pH 7.0 (▪). Protein concentration was 40 µM. Other experimental details are reported in Materials and methods. **Panel B**: Electron microscopy image of the protein in the presence of 0.1 mg/mL heparin at the beginning of the aggregation process. **Panel C**: Cell viability of NIH-3T3 cells exposed to wild-type apomyoglobin aggregates formed in the presence of 0.1 mg/mL heparin at pH 7.0 (grey) and at pH 5.5 (white) detected by MTT assay. Aliquots of protein were taken at 0, and 6 days from the onset of the aggregation process and incubated for 24 h with cells. Data are expressed as average percentage of MTT reduction ± SD relative to cells treated with medium plus heparin, from triplicate wells from 5 separate experiments. Other experimental details are reported under “Materials and Methods”.

We monitored the structural evolution of the aggregates using FTIR spectroscopy, ThT fluorescence and electron microscopy. The FTIR spectra of the wild-type apomyoglobin at pH 5.5 in the presence of heparin recorded 2 days after the onset of aggregation showed an amide I' maximum close to 1625 cm^−1^ (data not shown), which indicates the presence of the cross-β structure. The ThT fluorescence confirmed this result. As expected, in the absence of heparin, fluorescence did not increase at either pH 7.0 or pH 5.5. Conversely, ThT reactive aggregates were readily formed in the presence of heparin (Figure 8A). Electron microscopy showed the presence of fibrillar structures (Figure 8B). Figure 8C shows the effect of the heparin-induced wild-type apomyoglobin aggregates on cell viability. Aggregates were added to the cultured cells at various times after aggregation onset. The protein aggregates formed at the start of aggregation at pH 7.0 killed about 60% of cells, whereas the aggregates formed at pH 5.5 were harmless. Six days after aggregation onset, the aggregates formed at both pH values were not cytotoxic. The different cytotoxicity of the aggregates formed at the start of aggregation at pH 7.0 and pH 5.5 could be related to their different compactness. At pH 7.0, the low number of electrostatic interaction between heparin and protonated hystidyl residues makes the aggregates less compact thereby determining an increase of their exposed hydrophobic area [61]. This different toxicity could also be due to the acceleration of the heparin-induced aggregation that occurs at pH 5.5 (Figure 7A). In fact, it could well be that under these conditions, a reduced steady-state level of early toxic aggregates is reached consequent to the increased rate of oligomer growth into harmless higher order assemblies, as recently reported for TTR aggregation in the presence of heparin [46].

Taken together, the results we obtained with wild-type apomyoglobin indicate that heparin is able to induce an amyloid aggregation process that readily terminates with the formation of a fibrillar species rich in cross-β structure.

### Effect of different glycosaminoglycans and polymers on aggregation kinetics

**Figure 9:**
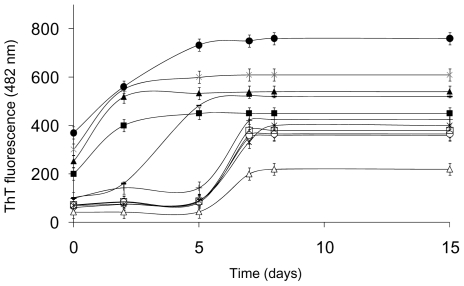
Effect of different GAGs on fibrillogenesis process of W7FW14F apomyoglobin at pH 7.0 monitored by ThT fluorescence. (◊) absence of GAGs; (•) 0.1 mg/mL heparin; (x) 0.1 mg/mL heparan sulfate; (▴) 1 mg/mL dextran sulfate; (−) 1 mg/mL condroitin A; (▪) 0.1 mg/mL low molecular weight heparin; (+) 1 mg/mL condroitin B; (*) 0.6 mg/mL polyarginine; (□) 1 mg/mL condroitin C; (○) 0.7 mg/mL dextran; (Δ) 0.6 mg/mL polylysine.

The magnitude of the effect of GAGs on amyloid aggregation depends on the GAG used [25], [29]. Therefore, we examined the effect of different GAGs and polymers on W7FW14F apomyoglobin fibril formation. As shown in Figure 9, similar to heparin, both heparan sulfate and lower molecular weight heparin greatly increased the yield and rate of fibril formation. Chondroitin sulfate A increased the yield and rate of fibril formation to a much lesser extent, whereas chondroitin sulfate B and C did not have any effect. We also examined the effect of a charged polymer, dextran sulfate, and found that it had, reproducibly, an effect similar to that of heparin. Therefore, also dextran sulfate accelerated amyloid fibril formation, which suggests that the presence of negative charges promotes amyloid fibril formation. To determine if the enhancing effect of dextran sulfate is due to the sulfates, we tested dextran, and found it had no effect. Therefore, the presence of negatively charged groups seems to be essential for the stimulatory effect exerted by charged polymers on the W7FW14F apomyoglobin mutant. As negative control, we tested the effect of the positively charged polyelectrolytes polylysine and polyarginine. Unlike polyanions that promoted the aggregation of positively charged proteins, the polycations did not affect the aggregation process.

## Discussion

It is generally agreed that amyloid fibril formation results from a nucleation and growth process and that the presence of a lag phase reflects the time required for nuclei to form [1]. The amyloidogenic W7FW14F apomyoglobin forms fibrils in not less than 9–10 days after a lag-phase lasting 4–5 days [48]. We found that, in the presence of heparin, mutant apomyoglobin forms branched and thicker fibrils within 2–4 days of the start of the aggregation process. This is mainly due to the electrostatic interaction between the positively charged side chains of apomyoglobin and the negatively charged regions of heparin that leads to the formation of protein-GAG complexes. This interaction may partially mask the charge–charge repulsion between the neighboring apomyoglobin molecules thereby determining a considerable increase in the local concentration of protein, which is a factor favoring rapid fibrillation [25]. Our experimental observation that heparin-induced acceleration of fibril formation is overturned when salts are added lends weight to this concept.

Given the strong dependence of lag phase on heparin concentration (Figure 4), we postulate that heparin templates the monomers or oligomers to associate thereby reducing the lag phase of the W7FW14F apomyoglobin fibrillization process. This hypothesis is substantiated by the finding that at the highest heparin concentration used, the aggregation rate in the early stage of the process was four-times higher than that observed in the absence of heparin, and the fraction of soluble protein was strongly reduced (Figure 1). Taken together, these results suggest that heparin promotes the formation of a higher concentration of nuclei starting from monomeric soluble species and/or oligomeric aggregates.

The FTIR spectra recorded soon after aggregation onset were consistent with rapid formation of cross-β rich nuclei acting as seeds for fibril formation. This conclusion corroborates the concept that heparin provides a spatially organized network that keeps protein molecules together thus allowing them to interact with each other [14], [62]. In this context, it is noteworthy that, at a low heparin concentration, 40 apomyoglobin molecules are bound to each heparin molecule (Figure 4, inset). The large molar excess of protein over heparin could also be indicative of the binding of heparin to oligomeric, rather than to monomeric forms of apomyoglobin. At increasing heparin concentrations, the ratio of apomyoglobin to heparin decreases from 40 to 5, which is consistent with the possibility that heparin binds monomeric instead of oligomeric forms of apomyoglobin. However, we can not exclude that, at the highest concentration used, heparin might induce conformational changes leading to a more extended, ordered spatial orientation of protein molecules in the early formed aggregated complexes. The binding of heparin to amyloid proteins has been reported to increase the degree of order of the protein within the aggregates, thus favoring the fibrillation process [32].

**Figure 10:**
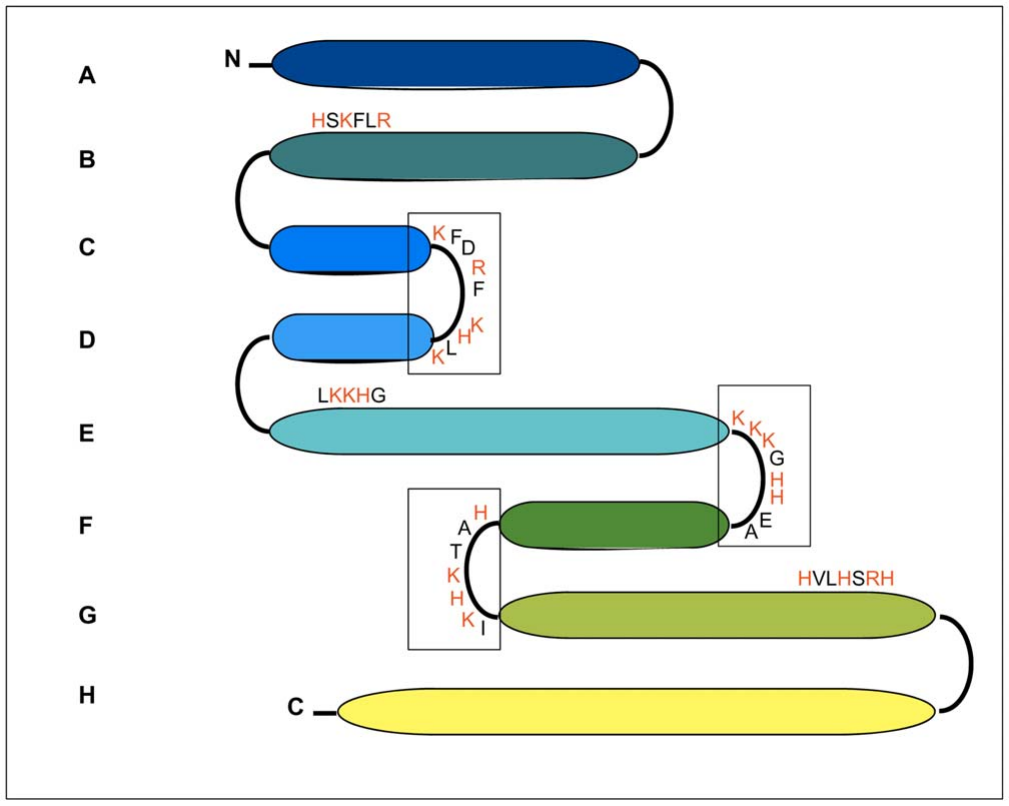
Consensus sequences for heparin binding along the helices in apomyoglobin structure. Each of the 8 alpha-helices is marked with a letter and is represented by an ellipsoid of size proportional to the length of the sequence. Residues corresponding to Cardin and Weintraub motifs are boxed. Basic residues are shown in red.

In a variety of proteins that are induced to form *β*-structure, heparin binding sites have been shown to contain clusters of basic amino acid residues capable of binding to the negatively charged heparin molecules [63]–[66]. In particular, Cardin and Weintraub [63] reported that heparin binding domains usually contain the consensus sequences *X*BBB*XX*B*X* or *X*BB*X*B*X*, where B is a basic amino acid and *X* is a non basic amino acid in both the alpha-helical and beta-strand conformation. Apomyoglobin contains three consensus sequences corresponding to the above-indicated sequences, localized in the turn regions between helices C–D, E–F, and F–G. Moreover, clusters of basic amino acids that do not conform to the sequences identified by Cardin and Weintraub are present in the primary structure at the end of the B helix, i.e., RLFKSH, the beginning of the E helix, i.e., LKKHG, and at the end of the G-helix, i.e., HVLHSRH (Figure 10). We propose that the crucial step of heparin-induced fibrillation is the recognition and binding to the basic cluster sequences localized on turn regions of the protein that are easily accessible. The observation that heparin induces amyloid aggregation of both partially folded and fully unfolded apoprotein suggests that the basic binding sequences are not necessarily located in helical structured regions. Heparin can, therefore, act as an immobilizing charged surface on which protein monomers are correctly oriented for priming an ordered polymerization that generates fibril seeds. It is also possible, as documented by our FTIR analysis, that heparin directly promotes the α-to-β structural transitions within monomeric species of the W7FW14F apomyoglobin mutant and so lead to acceleration of aggregation and fibril formation. At present, it is difficult to determine whether heparin simply acts as a concentrating surface on which protein molecules become in close contact or whether it induces conformational transitions as a consequence of which the formation of cross-β structure is favored. Probably, both effects are responsible for the observed acceleration of the aggregation process. Finally, we can not exclude that heparin induces a pathway of aggregation different from that of the amyloidogenic apomyoglobin in experimental conditions similar to the physiological setting.

The proposed mechanism for the heparin-induced aggregation and fibrillation of the W7FW14F mutant under aggregating, i.e., pH 7.0, and non-aggregating experimental conditions, i.e., at pH 4.0 and 2.0, is supported by the results obtained with the wild-type apomyoglobin, a protein that does not undergo aggregation and fibril formation under native conditions. In fact, we found that the addition of heparin to this protein caused aggregation and fibril formation that was much more evident on lowering the pH from 7.0 to 5.5. The increased susceptibility to aggregation after pH lowering could be due to histidine protonation, which increases the net positive charge of the apomyoglobin thereby favoring the heparin-protein interaction. However, it can not be excluded that pH affects protein stability. It has been shown that lowering pH from 7.0 to 5.5 reduces the conformational stability by about 2–3 kcal/mole thereby making the protein more susceptible to perturbing agents [67], [68]. The observation that the addition of salt at pH 5.5 does not influence the heparin-induced aggregation profile indicates that the increased aggregation is not only related to histidine protonation but also to a greater propensity of the protein to undergo structural modifications. In this context, the proton gradient formed in proximity of the heparin surface is likely to modify the structural properties of the protein and possibly favor its misfolding.

We also show that the effect on W7FW14F apomyoglobin fibril formation varies depending on the polymer used. A similar variability has been reported for other amyloidogenic proteins. In the case of α-synuclein, heparin and dextran sulfate had a stronger effect than polyglutamic acid, chondroitin-4-sulfate and dermatan sulfate, whereas chondroitin-6-sulfate and inhibition by dextrane had little or no effect [29]. Our results with mutant apomyoglobin show that acceleration of fibril formation depends on the polyanionic character of a GAG. In fact, neutral compounds such as dextran or positively charged compounds such as polylysine or polyarginine did not stimulate fibril formation.

The fibrillization-accelerating effect of heparin has interesting therapeutic implications because it induces the protein to assume a non-toxic fibrillar conformation. Increasing aggregation rates reduce the lag time for fibril formation, thereby reducing the time of exposure to toxic intermediates that cause cell death. In fact, we show that the protein is harmless when incubated with heparin. The most interesting aspect of our study is that heparin induced amyloid formation of natively folded wild-type apomyoglobin, which normally does not show any tendency to aggregate. Thus, heparin could play a dual role in amyloidosis, as a safe compound and as a pathological chaperone. The question is whether GAGs can be used as a therapeutic target in patients affected by amyloid-associated diseases or whether they are deleterious to health because they increase the fibril load. In fact, promoting formation of intracellular amyloid inclusions in Parkinson's and Huntington's disease may protect against pathological damage [69], [70] or may enhance oxidative stress by promoting cell death [71].
